# An Expose of Opium “Antidotes”

**Published:** 1877-06

**Authors:** J. B. Mattison

**Affiliations:** Brooklyn, N. Y.


### An Expose of Opium "Antidotes."

By J. B. Mattison, M. D., Brooklyn, N. Y. Read before the Medical Society of the County of Kings, Feb. 20, 1877.

At the request of the President of this Society, I have the honor of presenting an expose of a reputed "opium antidote," gotten up by a certain S. B. Collins, of La Porte, Ind.

I have recently been in correspondence with a gentleman, not unknown to literary fame, who, induced by the demands of a painful neurotic disorder, has for years been an opium habituate. What with that desire for relief which prevades suffering humanity, that distrust of self, and hesitancy to accept regular, scientific treatment, so eminently characteristic of such unfortunates, and lured by the laudatory assertions of this individual, he allowed himself to be enveigled into his hands, and after several months "treatment" awoke to the fact that he was still "in the bonds," and his exchequer depleted to the extent of nearly two hundred dollars! This gentleman very kindly furnished me with a liberal supply of this so-called "antidote" which I placed in the hands of Dr. Squibb, for analyzation, to whom I return my thanks. Dr. S. being prevented from making the analysis in person, submitted it to chemists in New York, in whom he expresses confidence, who reported:—

"Result of analysis of an 'opium antidote,' received from Dr. E. R. Squibb.

| | |
|---|---|
| Water, | 28.66 |
| Glycerine, | 66.89 |
| Cryst Sulphate of Morphia, | 4.45 |
| | 100.00 |

The sample is colored with magenta.

WALZ & STILLWELL."

This proportion of morphine amounts to *twenty-five and a half grains to the ounce!!*

Mr. Stillwell informed me that it contained nothing else in appreciable quantity.

Additional evidence is furnished by the analysis of another sample of this nostrum, made last Autumn, by Dr. Henry Carmichael, Professor of Chemistry, Bowdoin College, and Assayer to the State of Maine, who reported:

"The 'opium antidote' contains morphine,

"The morphine is combined with Sulphuric Acid,

"The Sulphate of morphine amounts to 3.2. per cent., or *fourteen grains to the ounce"!*

The same, precisely, applies to another compound prepared by "Mrs. J. A. Drollinger, formerly Mrs. S. B. Collins."

Last August, a specimen of her manufacture came into the possession of Dr. George F. French, of Portland, Maine, who brought it before the Cumberland Co. Med. Soc., which appointed a Committee to investigate. They reported:

"The Committee to whom was assigned the duty of investigating the so-called 'Opium Antidote' prepared by Mrs. J. A. Drollinger, of La Porte, Ind., beg leave to report that a sample bottle of the article, which was obtained directly from the manufacturer, was sent to Dr. E. R. Squibb, of Brooklyn, N. Y., for quantitive analysis. His onerous engagements rendered it impossible for him to conduct the investigation in person, but he sent the specimen to Messrs. Walz & Stillwell, Chemists, New York, a firm which he thoroughly confides in and endorses. So deeply interested did he become in the project, that he insisted upon bearing the expense of the analysis, in spite of the Committee's expressed unwillingness to have him assume such a tax. Walz & Stillwell report that 'this sample is glycerine colored with analine red, and containing in solution Crystalized Sulphate of Morphia, 1.383. per cent. by weight'—about *seven grains to the ounce!*

Frederick Henry Gerrish,
George F. French,
Thomas A. Foster,
Committee."

Further corroborative testimony is to be found in an interesting paper by Prof. Stanford B. Chaille, in the N. O. Med. Jour. May, 1876, regarding a gentleman who, beguiled by another of this fraternity,—J. C. Beck, of Cincinnati, had for nearly a year, been taking his nostrum, without relief, and was at last rescued by Dr. Chaille, who, with judicious treatment, restored him to health in a comparatively short time. A sample of this submitted to Mr. J. Johnson, Chemist of N. O. Charity Hospital, revealed *ten grains of Opium to the ounce!*

Collins claims to be the pioneer in this business. Since his advent, several others have come to the surface. Indiana has the van, furnishing no less than six—Collins, Drollinger, L. Meeker, D. Meeker, Bowser, Squire; Ohio, two—Beck, Wilford; Illinois, two—Carlton, Phelon; Michigan, one—Marsh. Recently, one has appeared in New York, and another in New Jersey.

I possess all their circulars, with a single exception. Their pretensions are preposterous; their falsehoods glaring.

Collins says "it is not a substitute for opium. That opium does not and cannot antidote itself, is a sufficient reply to your second query."

Drollinger avers "it does not contain opium in any of its numerous forms." Beck asserts "it has no opium in it." The *absolute falsity* of such statements, in view of the analyses presented, is too patent to call for comment.

Regarding *modus operandi*, the article is supplied in pint bottles, each supply intended to last a month. The manner of taking is a small teaspoonful four times daily—8 and 11 A. M., 3 and 8 P. M. None is furnished without a cash payment in advance, and after that at prices ranging from five to thirty dollars per month, according to the amount of opium previously consumed, and—*the facility with which they succeed in fleecing their victim.*

Such is the history of these nostrums and their venders. The wide-spread importance of the topic is obvious, and it is to be hoped this information may be given a large publicity, that, through the profession and others, opium habituates may be "warned against these widely advertised nostrums, which, while promising a safe and sure cure, only serve to increase the appetite for opium, until it finally becomes unconquerable."

